# A Warning to the Government

**Published:** 1919-08-16

**Authors:** Henry C. Burdett

**Affiliations:** The Lodge, 13 Porchester Square, W. 2


### THE AFGHAN FRONTIER.

### HEALTH OF THE TROOPS.

### A Warning to the Government.

Reprinted from the *Times*, 12/8/19.

Sir,—The Secretary of State for India's written answer to a question by Colonel Yate concerning the arrangements made for the health and comfort of the troops on the North-West Frontier of India, published in the *Times* of the 6th inst., though lengthy, is incomplete, and omits the most material point of all.

Nothing is said of the medical organisation, but the conditions for our forces, especially in the fighting line, are very bad—great heat, great dust, and a rough, uncivilised country. There is, I believe, an excellent motor-ambulance convoy, the officers and men of which are all European and are thoroughly reliable. It is in view of the presence of cholera, recently reported, and that the medical *personnel* with the regiments and field ambulances were, and probably are to-day, as to the larger percentage Indian, and not qualified Indians, but a considerable number of them men holding temporary commissions in the I.M.S., whose only training would have been in the Indian Medical Schools, that I feel it a patriotic duty to ask the Secretary of State to make specific inquiry as to the actual strength and quality of the medical organisation prevailing with the troops now opposed to the forces of the enemy in India at the present moment. The Indian sections just mentioned do not consist of qualified medical men in the English sense. Their qualification is not registerable. They could not, for example, even work as assistants to a panel doctor in England, and would be liable to prosecution if they described themselves as qualified men in England. Is it true that these men with the Army have been put on an equality with the men of the R.A.M.C. and I.M.S. for the purpose of treating the sick and wounded? A very few of them just possibly may have the requisite knowledge, but the majority do not possess it. To show on paper such an Indian I.M.S. as its regimental surgeon is a grave scandal, for in reality such a regiment can have no properly qualified medical man at all, but only a man who has had a training of sorts at an Indian Medical School, and has a good diploma to practise which only holds good in India. Such men have seldom any power of command, and are therefore not good at keeping men up to the mark in camp sanitation, even if any of them possess a good knowledge of the subject, which they seldom do.

I have good ground to fear that when we hear it reported from India that an Army Division has three field ambulances with it, it probably means what in India is merely called a combined field ambulance, that is, one divided into four sections—a British and three Indian. Now, instead of such a section being the equivalent of a section in the field ambulances working in France and Macedonia, which proved themselves extraordinarily strong units for the officers in command to deal with, these combined field ambulances in India appear to be made by taking a section from something that is called a British field ambulance and three sections from what is called an Indian field ambulance, and so making a unit which is called a combined field ambulance. In such a case there is no headquarters establishment, and each section has only one commissioned medical officer. Of the commissioned medical officers usually two will be there who are in reality unqualified Indian medical men; the remaining assistant surgeons attached belong to what was formerly called the Indian Subordinate Medical Department, a useful body that works in the Army hospitals of India under their qualified medical officers. The best of them are something of the type of the old unqualified assistant. There are also men called assistant surgeons, who are seldom of any use except to dress a minor injury or to do some dispensing.

The nursing establishment is represented by four half-trained ward orderlies, British or Indian, who probably could not pass a V.A.D. examination or St. John Ambulance examination.

We have good cause in England to fear that the grave abuses which prevailed in Mesopotamia may only have hardened the hearts of the Indian Government, as Pharaoh's was hardened, hence the deficient medical arrangements for our soldiers in India, which existed to my knowledge within the last three months. The defective, inefficient, and improper medical equipment I have described can only have arisen because the Indian Government would not find the money to bring the medical arrangements for our soldiers up to a proper standard. We at home, grateful as we are to our splendid soldiers, cannot suffer any repetition of the scandals of Mesopotamia to occur on the North-West Frontier of India, and I hope and believe, if it still does prevail, and some of the first born or highest born are destroyed by a scandal even greater than that of Mesopotamia, that a wave of indignation will properly be raised that will insist upon the removal and dismissal from the Service of those connected with the Indian Government who may be found responsible. This grave matter presses, and I rely upon Parliament bringing such pressure to bear on the Secretary of State for India that adequate and ample medical organisation and equipment, both in men and material, may be sent forward without a moment's delay to bring the medical arrangements for our soldiers up to a full and proper standard.—I am, Sir, yours faithfully,

Henry C. Burdett.

The Lodge, 13 Porchester Square, W. 2,
August 8, 1919.

